# Effect of Educational Interventions on Understanding and Use of Nutrition Labels: A Systematic Review

**DOI:** 10.3390/nu10101432

**Published:** 2018-10-04

**Authors:** Sally G. Moore, Judy K. Donnelly, Steve Jones, Janet E. Cade

**Affiliations:** 1 School of Food Science and Nutrition, University of Leeds, Leeds LS2 9JT, UK; J.e.cade@leeds.ac.uk; 2 School of Health and Social Sciences, Leeds Trinity University, Leeds LS18 5HD, UK; judykdonnelly@gmail.com (J.K.D.); st.jones@leedstrinity.ac.uk (S.J.)

**Keywords:** food labels, nutrition labels, nutrition education, nutrition knowledge, nutrition facts label utilization, nutrition facts label comprehension, health literacy

## Abstract

The potential for nutrition labels to impact on population health is dependent on consumer ability to understand and use this information. Consumer understanding of this information varies across sociodemographic groups and with different label design formats. Labeling legislation requires consumer education on how to use nutrition labels, and recent mandatory changes to the Nutrition Facts Panel (NFP) are underway to improve comprehensibility. This review aimed to evaluate if educational programs can improve understanding and use of nutrition labels. Database searches were performed to identify interventions which delivered education on nutrition labels with outcomes measuring aspects of comprehension or use. A total of 17 studies were selected for review, including nine randomized and eight cohort studies. The majority of studies were conducted in the United States Study participants included school aged children, older adults, and those with diabetes within a range of intervention types involving taught sessions or web-based education. Whilst outcome measures were heterogenous, all studies reported a statistically significant improvement in one or more outcomes of participant understanding or use of nutrition labels. Aspects such as general nutrition knowledge, health literacy, and program delivery format warrant attention in future research. Education which optimizes comprehension and use of nutrition labels may have the potential to improve the impact of this information on dietary health.

## 1. Introduction

Nutrition labels display information about the nutrient content of food and drink products and are intended to guide healthy food choices. Consumer use of this information varies, but it is estimated that around 50% of consumers report reading this information [1]. Using nutrition information to shape healthy dietary choices requires consumer understanding and interpretation of nutrient contents and dietary recommendations [2]. Therefore, an individual’s understanding or “knowledge” of what nutrition label information means should theoretically precede consumers’ actual use of this information in evaluating food purchases [2]. However, understanding of nutrition label information has been found to vary across different age groups, genders, and educational attainment levels [1]. In addition, it is known that adequate health literacy, defined as the ability to obtain, understand, and use basic information needed to make decisions about health [3], as well as numeracy, are key characteristics associated with use and understanding of this information [4]. As such, there is likely to be a disadvantage for some consumer types, including those with limited levels of numeracy and health literacy, who are expected to use this information to make healthy choices. 

Reviews of consumers’ use and understanding of nutrition labels have consistently highlighted lack of understanding as an important barrier to use of this information [1,5]. Specific comprehension difficulties include consumer understanding of quantitative information and interpreting correct serving sizes as well as the recommended % daily values (%DVs) [1]. For example, the majority of consumers appear to be able to locate calorie content on the Nutrition Facts Panel (NFP) [6], yet fewer are able to use percentage information or serving size data to estimate the contribution to the daily diet [7,8]. Consequently, research attention has been focused on how changes to the format of nutrition information may impact on consumer understanding, including additional front-of-pack signposting (e.g., traffic light) schemes [9,10]. Legislation and public health initiatives have recommended label format changes to improve comprehensibility of nutrition information in order to make healthy choices easier for consumers. Such modifications include the display of calories and added sugar as part of the NFP [11,12], and the specific format of front-of-pack nutrition signposting in the United Kingdom (U.K.) and France [13].

Provision of education to promote use of nutrition labels is included in the Nutrition Labeling and Education Act in the United States (U.S.) [14], as well the European Regulations on Food Information for Consumers [15]. This legislation makes nutrition labeling mandatory on food products. Modeled predictions of the impact of providing such mandatory nutrition information have shown the potential to decrease obesity in Europe by 2.5% [16], assuming consumers receive explanatory information about how to read labels [17]. Slightly greater improvements to health are predicted with label education as part of individual counselling by a combination of dieticians and physicians [17]. Therefore, from both legislative and theoretical perspectives, there exists a clear role of education to enhance the efficacy of nutrition labels on health improvement. 

Calls for consumer education to increase understanding of labels also emanate from research reporting the mixed and disappointing impact of simply displaying nutrition labels on food products. For example, review evidence has reported a lack of impact of on actual purchase behaviors of nutrition labeling or product health information displayed at the point of purchase [18]. Additionally, a meta-analysis of nine studies assessing the impact of both mandatory and various front of pack nutrition labels suggested such labeling could result in more people selecting healthier food products in those experimental and real-life settings and decrease calorie choice/intakes by 3.5% [19]. The extent to which education can optimize nutrition label use is not yet known, but emerging evidence indicates that displaying explanatory signage in-store may improve consumer use of this information and their selection of healthier choices [20].

There is a need for insight into if and how the efficacy of nutrition label information can be enhanced by consumer education. No review has yet examined the effect that educational interventions may have on consumer understanding and use of nutrition labels. This review aims to describe the effect of such interventions on nutrition label use and understanding. It also aims to provide an examination of the literature on the design features of these programs to inform further research and intervention evaluation.

## 2. Materials and Methods

This review was undertaken in accordance with the Preferred Reporting Items for Systematic Reviews and Metanalyses (PRISMA) guidelines (Supplementary Information S1) [21]. 

### 2.1. Search Strategy and Study Selection

The electronic databases Medline, PsychInfo, and Cinahl were searched for records published between 1994 to March 2015 (search 1) and again between April 2015 and July 2018 (search 2). The year 1994 was chosen as the earliest publication date to encompass educational programs occurring following the implementation of the U.S. Nutritional Labeling and Education Act. Search terms and strategies were created using key words from previous literature and database-specific subject headings to identify studies evaluating the effects of nutrition label education interventions on the outcomes of consumer use and knowledge (understanding) of labels. Search terms were combined using three elements of the research question (e.g., “nutrition label information” or “nutrition facts panel” AND “educational intervention” or “education program” AND “comprehension” or “use” or “knowledge”) (see Supplementary Information, Table S1). To ensure the results reflected the aims of this review, abstracts were screened for articles in English reporting interventions which included nutrition label education either alone or as a component of a wider multi-component program and evaluated outcomes which specifically included use or understanding of nutrition label information. Following screening of abstracts, full text articles were obtained and assessed against the specific exclusion criteria by the first and second authors, independently, with subsequent discussion to resolve conflicts. Exclusion criteria were study types including: (A) no outcomes concerning nutrition label use or understanding, (B) examination of the impact of different formats of nutrition labels on consumer comprehension since these have been reviewed elsewhere [1], (C) education on “food labels” involving aspects of the label which were not nutrition information such as allergen or ingredient information, and (D) evaluations of implementation of labeling on products or “healthy eating” in-store campaigns (without educational sessions) including measures of nutrition or health literacy using nutrition label “quiz” instruments. No studies were excluded based on their geographic location, labeling format, target audience, or study design.

### 2.2. Quality Assessment and Data Extraction

A total of 17 studies were included and appraised for quality by the first author in discussion with the research team using the Effective Public Health Practice Project (EPHPP) assessment tool for assessing risk of bias in intervention evaluations [22]. Each of seven study characteristics including study design, participant selection, and attrition were rated as “weak”, “moderate”, or “strong” based on the potential for bias and EPHPP ratings. Where quality EPHPP criteria aspects were not clearly reported, further information was sought from the study authors by email. If no response was obtained these items were rated weak after discussion with the research team. Two study authors were contacted concerning intervention content or evaluation measures in order to assist the data collection process; however, no responses to these requests were obtained. In line with the research objectives, data extracted from the studies included participant and intervention program characteristics, as well as descriptions of, and impact on, outcome measures concerning the use and understanding of nutrition label information. 

## 3. Results

**Figure 1:**
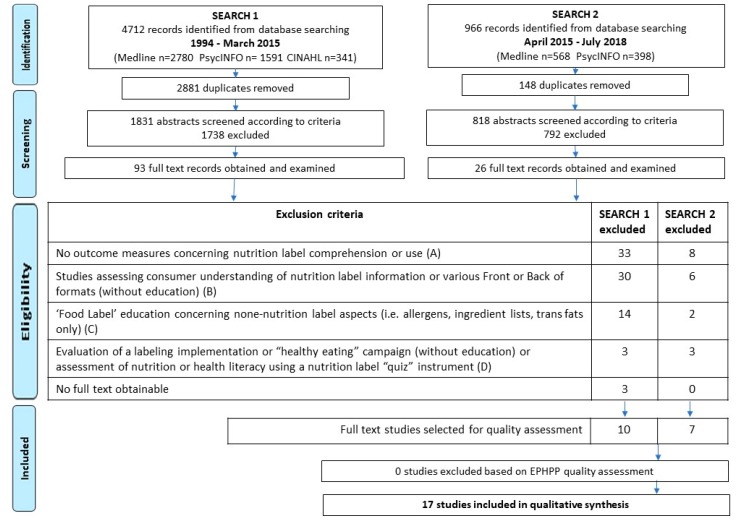
Study selection of articles included in the review. EPHPP: Effective Public Health Practice Project.

Database searches 1 and 2 conducted in 2015 and in 2018 returned 4712 and 966 records, respectively (see Figure 1). Following duplicate removal and screening of abstracts, full texts (119 in total across both search timeframes) were examined in detail against the exclusion criteria. For example, 41 studies which involved aspects of nutrition label reading in their educational interventions but did not evaluate label use or comprehension as outcomes were removed. Designs of the 17 included studies were eight cohorts and nine randomized studies, of which three used comparator intervention-receiving groups and six used control groups. Overall quality of the 17 studies was appraised as “moderate” for ten studies, “strong” for five studies (see Supplementary Table S2), and “weak” for two studies due to acknowledged limitations concerning confounding or very low numbers of participants at follow-up [23,24]. No studies were removed due to EPHPP quality rating so that all 17 studies were retained for onward qualitative synthesis in this review to provide an inclusive analysis of interventions undertaken in different settings.

### 3.1. Intervention Participants and Programs 

The final selected 17 studies included a total of 5421 participants, which were >50% female, and entirely female in one study [25] (Table 1). Ages of participants ranged from the third grade (8 years) [26] up to 75 years old [24,27]. Participants included university students [28,29], school children or adolescents [26,30,31,32,33], disadvantaged or vulnerable adults [34,35] on existing education programs [36], or low-income adults [37], some with low health literacy [38]. Four studies were specifically conducted with adults with diabetes [25,27,35,39]. Most interventions took place in the United States (*n* = 12) and Canada (*n* = 2) prior to 2017, with the remaining three from India, Australia, and the United Kingdom.

Almost half of the interventions (*n* = 7) focused on nutrition label education entirely in a one-off session (Intervention Type 1) [23,24,28,29,30,31,33,38]. The shortest intervention was a ten-minute booklet viewing session [28]. The remaining 10 programs promoted nutrition label use and understanding skills as part of a healthy eating intervention (Intervention Type 2) [25,26,27,32,34,35,36,37,39]. These included sessions delivered to groups in community or school settings weekly [25,27,32,34,36,37], or monthly [39], with participants in one study receiving individual intensive home-based visits as part of a 12 month intervention [35]. Across both intervention types, delivery formats included web-based education conducted with individual participants in two studies [29,37] and interventions conducted entirely [24] or partly in a supermarket [26,27,35].

### 3.2. Effect on Understanding of Nutrition Labels 

The 11 studies which evaluated participants’ understanding or “knowledge” of nutrition labels reported statistically significant pre-post intervention increases in this outcome in their cohorts [24,29,30,33,36,39] or relative to the comparison group [25,26,27,31,38] (Table 1). Considerable variation existed in questions used to evaluate understanding of nutrition labels at pre and posttest. Studies which conducted extensive evaluations of participants’ own understanding of nutrition labels used multiple quiz questions assessing ability to interpret and compare labels on serving size, and nutrient content information to show positive effects of their single education sessions on pre-posttest scores [33,38]. Other studies used only a few questionnaire items on nutrition labels which were included among those concerning other food label information involving ingredients, quality logos, cooking instructions etc. [23,24,31,34,36]. In one case, change in “knowledge of the nutrition label” was assessed using a single question asking if nutrition information was “present” on a food label [31]. 

Two studies used a validated multi-item “food label literacy” instrument to evaluate ability to use nutrition labels and make healthful food choices. Both showed significant pre-posttest improvements among the school children undertaking the intervention [26,30]. Accuracy of use of nutrition label information was evaluated objectively in a study with undergraduate participants who undertook repeated web-based “training with feedback” by working through several pairs of nutrition labels to identify the correct healthy choice [29]. This intervention was found to significantly increase participants’ nutrition label reading skills in terms of such accuracy, as well as decreasing the time taken to evaluate labels. 

Aspects of both factual and applied knowledge and understanding of nutrition labels were evaluated in two studies with participants with diabetes [25,27]. These participants undertaking multi-session programs increased pre-post intervention measures of both declarative (i.e., factual) and procedural (i.e., applied) knowledge of nutrition labels. In addition, one study was also able to quantify improvements in participants’ own decision-making rationale for theoretical food purchases [27]. This study highlighted the potential for enhanced ability to use numerical nutrition label information to make specific product comparisons and choices in the context of diabetes management [27]. 

### 3.3. Effect on Usage of Nutrition Labels 

There were 13 studies which evaluated impact of their interventions on nutrition label “use”, with all showing significant improvements in one or more measures of this outcome (Table 1). Use of nutrition labels was evaluated using mainly self-reported pre-post questionnaire items [31,32,35,36,37] (i.e., “*How often do you read nutrition labels?”*). Objective use was evaluated in one study which found a significant increase in eye gaze time (by 1.3 s) in those viewing nutrition labels compared to the control group following a brief leaflet-viewing intervention [28]. Levels of self-confidence in using labels, including for specified tasks (i.e., “*I can use nutrition labels to check sugar content*”) improved significantly following intervention in four studies [24,25,27,34], as did perceived importance of reviewing this information before purchase [23]. One study with school children used five questions to assess nutrition label use which included “*Do you see the sugar content in sparkling beverages?”* and “*Do you see the salt content when buying snacks*?” [31]. Of these, children’s responses to only the latter question were significantly improved in the intervention compared to the control group. 

Three studies found evidence of increased use of nutrition labels at follow-up, which took place sometime after the final intervention session [34,35,36]. This included at 6-week follow-up with 927 disadvantaged Australian adults [36], and after 3–4 months with 62 vulnerable Scottish adults [34]. In addition, participants with diabetes who had individual, multiple home-based sessions over a 12-month intervention reported significant impact on frequency of use of nutrition labels up to 6 months after the intervention (i.e., 18 months after the start) [35]. However, due to the considerably reduced number of participants which returned for follow-up, the risk of bias should be noted.

## 4. Discussion

The purpose of this review was to systematically examine the effect of educational interventions on participants’ use and understanding of nutrition label information. All studies reviewed here were effective in improving one or more measures of use or understanding of nutrition labels with a variety of participant types and settings and delivery formats.

### 4.1. Design Features of Effective Interventions 

The 17 interventions reviewed here can be categorized into two intervention types. These are Type 1: those focused entirely on nutrition label education mostly during a one-off program or session [23,24,28,29,30,33,38], and Type 2: multi-component programs encompassing nutrition label education alongside other aspects including behavioral components such as healthy cooking and lifestyle advice [26,31,32,34,36,37] and also in the context of diabetes management [25,27,35,39]. Type 2 studies include interventions designed around theoretical models of behavior change such as “Social Cognitive Theory” and, “Stages of Change” [39]. In terms of nutrition label education, underpinning theories across both intervention Types 1 and 2 include theories of Kolb’s experiential learning [25,32,37,39], information processing [27,28] and skill acquisition [29]. Whilst theory-based interventions are often recommended to promote behavior change [40], both intervention Types 1 and 2 can be seen to produce positive effects on the two outcomes of interest: use and understanding of nutrition label information. However, evidence of lasting follow-up effects was only gathered and found in the multi-component Type 2 interventions. 

Studies reviewed here provide some insights into the influence of the role of delivery format on the effectiveness of the intervention. A web-based intervention was conducted with low-income participants and compared with an in-person taught comparator group, both of which received three sessions of a healthy eating education program [37]. In response to the question “*When shopping do you use nutrition facts labels to decide what food to buy*?” this study showed significant pre–post intervention gains for both web-based and the taught in-person groups. However, these gains were greatest for the in-class taught group. Whilst the precise content of each delivery format was not described, it is possible that the more favorable results for the in-person taught group were due to providing in-person opportunities for participants to ask specific, personalized questions and to check their own learning and assumptions [40].

Another study incorporated technology (a multi-media video) into in-class sessions and was successful in improving nutrition label comprehension test scores in the intervention vs control group (who received reading materials). However, this intervention appeared ineffective for the small sub-group of outpatients identified as having low health literacy in this study [38]. Adequate health literacy has emerged as an important factor in use and understanding of nutrition label information [4]. Levels of health literacy have been shown to decrease with age, educational attainment and income [41]. Patients with lower levels of health literacy have been found to spend more time viewing non-relevant (nutrition label) information than those with higher levels [42]. It has been noted that an individual’s level of health literacy and nutrition label comprehension are often related since they are measured with similar tests involving nutrition label quizzes [4]. However, it is considered possible that improvements in health literacy may also enhance consumers’ understanding of nutrition label information [4,43].

Several interventions were described as devised or adapted to meet the needs of participants [32,34,36,39] or else targeted improvements in diabetic glycemic control [35]. In one case the intervention content was devised using research evidence showing “knowledge gaps” with food labels in people with diabetes [27]. These interventions reported improved use [32,34,35] and understanding [27,36,39] of nutrition labels in their participants, including those with type II diabetes. One study found participants in a home-based 12-month intervention were reported to improve their use of nutrition labels even after 6 months of follow-up [35]. In addition, these participants also improved their glycemic control, mediated via improvements in participants’ dietary intakes [35]. Aside from the specificity of the program content, the success of these interventions may also be due to participants’ inherent motivational and diabetic health-concerns, factors also known to drive nutrition label use [44].

The success of these interventions in improving understanding of nutrition labels may be attributed to their focus explaining the meaning of specific numerical elements of the presented information, such as nutrient content per serving and the percent “daily values” (%DV). Where intervention content on “nutrition label reading” was explicitly described in the studies, this included emphasis on %DV [24,30,33,38], serving size [35], and nutrient content per serving [25,27,29,31]. These elements have previously been reported to cause difficulties for consumers, amounting to barriers to understanding [1,5]. However, these explanations will depend on the label type used. For example, the U.S. NFP labels used in these studies were those prior to the changes now being implemented following the 2017 Food and Drug Administration modifications to the Nutrition Facts Panel. Also, whilst most studies were conducted in the United States and Canada, three studies were undertaken in India, the United Kingdom, and Australia. These will have used nutrition labels which were different to the U.S. NFP which shows per serving nutrition information and %DV amounts. For example, nutrition labels in the United Kingdom display nutrient content data “per 100 g” with additional information per serving, requiring explanation on how to use both of these elements during educational sessions [45]. Whilst this review did not compare interventions based on label format, it is important to note that detailed educational content including specific label elements and terminology should be guided by legislation on country-specific nutrition label formats. 

Studies reviewed here suggest it may also be possible to enhance the impact of focused nutrition label education on participants’ own understanding and use of this information with additional educational context concerning general or “healthy eating” nutrition knowledge. This can be seen in a single session intervention in which those participants receiving an in-class prior nutrition knowledge presentation were more likely to accurately assess nutrition label comparisons during online training than those who did not receive this presentation [29]. Levels of general nutrition knowledge have previously been associated with improved nutrition label use and understanding [2,46]. In terms of label use, significant post intervention gains in “belief in ability to use food labels” were positively associated with gains in tested “general nutrition and diabetes knowledge” [39]. Results from these studies also appear to agree with recent review evidence describing relationships between use and understanding of the nutrition label and levels of health literacy and numeracy [32,34,35]. For example, one of the studies reviewed here demonstrated a link between assessment of pre-intervention “nutrition label numeracy” and nutrition label task accuracy [29]. Nutrition knowledge and (nutrition label) numeracy may be important targets or considerations in future educational interventions concerning nutrition labels. Similarly, it is possible that education on nutrition labeling is a requirement to help reduce the existing inequalities in access and use of mandatory nutrition information as a means to improve health [43].

### 4.2. Outcome Measures

Strong evaluation of interventions relies on pre and post assessment and valid instruments and measures [47]. Considerable heterogeneity exists in the type and number of questions asked in pre and post questionnaires to evaluate objective understanding of nutrition labels which precluded metanalysis. Two studies evaluated participants’ understanding of the nutrition label element of the overall “food label” using a single question. These questions required participants to identify whether a nutrition label was present [31] or asked “*In 100 g of this product how many grams of sugar are there?*” [36]. In contrast, other studies used multi-item questionnaires focused entirely on nutrition label comprehension [38]. All studies reviewed here used pre and posttest measures, yet it is not clear to what extent these questionnaires themselves influenced participant understanding and therefore the validity of the results. For example, completion of questionnaires before and after the education sessions may have supported participants’ learning by increasing self-awareness about which aspects they did and did not understand. It is also known that claimed understanding of nutrition labels can be greater than objectively tested understanding [48]. Therefore, the role of self-awareness of understanding may deserve consideration in future interventions.

Usage of nutrition labels was mainly self-reported with indicators including confidence and frequency of use of this information. Self-reported measures are likely to be biased or over-estimated in terms of label use particularly in the context of the intervention setting. In addition, not all studies assessed both use and understanding of outcomes. In one case, where understanding was not assessed, this hindered full appreciation of the educational impact of the online intervention, which was found to impact less favorably on intended use of nutrition labels than in-person taught classes [37].

Whilst this review considered the outcomes of use and understanding of the nutrition label, it was not intended to assess any association between these outcomes. It has previously been theorized that understanding of nutrition label information is a key antecedent to its use by consumers in their decision-making processes during purchase choice evaluations [2]. However, it is important to note that even those who claim to use nutrition labels frequently may not fully understand this information [46].

### 4.3. Strengths and Limitations of the Review

The strengths and limitations of this review include a comprehensive search strategy and systematic selection process, undertaken on two occasions to ensure the most up to date publications were included and that inclusion criteria were rigorously applied. However, it is also possible that some relevant articles were not included in the review due to the number of databases searched. This review was global and the studies included spanned two decades, but only included five studies which were conducted outside the United States, demonstrating the potential need for future research in other countries with different label types. No unpublished grey literature was known nor searched and as such the risk of publication bias should be noted. Furthermore, statistical meta-analysis of effects of these interventions was not possible due to both heterogeneous study designs and outcome measures.

### 4.4. Implications for Practice and Research

Educational interventions with content concerning nutrition labels can been seen to have a positive impact on use and/or understanding of this complex numerical information. Research data is limited to a small number of studies, but these do include different ages and disadvantaged groups. The optimal setting and delivery formats of such education programs is not yet clear, but it is possible that these aspects depend on the needs of the specific target population. However, this review shows that there exists potential for even very brief one-off educational sessions to impact on understanding and use of nutrition labels across a variety of population types. The success of interventions in improving understanding of nutrition labels may be attributed to their focus on the specific numerical elements such as serving size and %DV, which have previously been reported as difficult for consumers to understand and use [1,5]. Practically, inclusion of behavioral and additional contextual general nutrition knowledge components which focus on dietary recommendations and healthy eating may further improve participants’ own understanding and use of this information. 

It is therefore possible that education on nutrition labels together with education on general healthy eating recommendations are important elements in interventions designed to impact on label use and subsequent food behaviors, including purchase choices [18,20]. The impact of current initiatives to improve nutrition label use, including legislation to enhance comprehensibility of this information, may also be enhanced by corresponding improvements to consumer understanding of this newly presented information. This review can be used to inform future development of educational initiatives aiming to increase the efficacy of mandatory nutrition label information. Future evaluation is needed to confirm if education which optimizes comprehension and use of nutrition labels has the potential to improve the impact of this information on dietary health.

## Figures and Tables

**Table 1:** Summary of studies reporting outcomes of “use” and “understanding” of nutrition labels.

Ref (Country)	Sample Characteristics (Size)	Intervention Program Description, Aims, and Delivery Format	Study Design, Follow-up (Control Group)	Setting	Session Duration	Theory	Nutrition Label Use		Nutrition label Understanding	
							Outcome Measure	Impact	Outcome Measure	Impact
[27] U.S.	Older adults with type 2 diabetes. 53% women. ≥65 years old. (*n* = 93)	Nutrition labeling education program to improve food label knowledge and skills in diabetes management. Delivered in-person by dietitians.	Randomized controlled trial. Pre-posttest. (Control group: no contact other than mailed questionnaire)	Outpatient	10 weekly group sessions (each 1.5 h)	SCT. IP	Confidence in using food labels. i.e., “*I can choose foods high in fiber”*	Significantly increased in experimental not control group (*p* < 0.001)	Total, procedural and declarative knowledge of the food label and decision-making skills.	Procedural, declarative and total knowledge scores and decision-making skills increased significantly for intervention group but not for control group (all *p* < 0.01).
[25] U.S.	Women with type 2 diabetes. 40–60 years old. (*n* = 40)	Nutrition labeling education program to facilitate the application of information on the food label to meet patient’s needs. Delivered in-person by dietitians	Pre-posttest control group design. (Control group: no contact other than mailed questionnaire).	Community centres	9 weekly group sessions	TML	Confidence in skills using the food label.	Significantly increased in experimental, not control group (*p* < 0.01)	Nutrition and food label related knowledge: Total, procedural, and declarative.	Both total, procedural and declarative knowledge were significantly increased in experimental, not control group (*p* < 0.01).
[38] U.S.	Low-income adult patients. Mean age 50 years. 73% female. (*n* = 42)	Intervention to improve nutrition label comprehension. Brief interactive multi-media video and pocket card. Tutor delivered.	Randomized (controlled) trial, Pre-posttest. (Control group: received black and white reading materials only).	Healthcare center	1 group session, 45 min total.	NS	Confidence in nutrition knowledge.	No significant difference between groups.	Nutrition label knowledge comprehension quiz score (%), including accurate interpretation of %DV and serving size information.	Quiz score pre-post gains were greater for the intervention group than the control group (*p* < 0.05). Sub group analysis of (*n* = 7) participants with low health literacy found no significant increase for either group.
[39] U.S.	U.S. older adults with diabetes. Mean age 63 years. 73% female. (*n* = 239)	Dining with Diabetes: Diabetes education program about healthy eating and food label components. Group taught sessions delivered in person.	Cohort using pre- posttests.	Community-based	3-monthly group sessions, 2 h each.	SoC SCT	Confidence in ability to use labels	Significant pre-posttest improvement (*p* < 0.01).	Knowledge questions included the nutrition label items. Exact details NS	Knowledge scores were significantly better post, compared to pretest (*p* = 0.001). Knowledge scores were a significant factor for response to “*Do you agree you know how to use food labels?*”
[24] Canada	Adults. Aged 31–75 years. >90% female. (*n* = 19)	An in-store Nutrition Label Education Program designed to teach how to read nutrition facts panel. Delivered by a registered dietitian using a lecture with materials followed by a store tour	Cohort pre- postsurvey and one-month follow-up (*n* = 3).	Grocery Store	A 2-h group session.	NS	Self-confidence, awareness and ability to use nutrition labels	Self-confidence performing all seven activities were significantly increased posttest (*p* < 0.01).	Self-reported knowledge of the NFP assessed using two items (serving size and definition of the term “percent daily value”).	Increase in number of participants answering %DV question correct (15.8% to 57.9%). Smaller increase in number of participants correctly identifying serving size (26.3% to 36.8%).
[36] Australia	Disadvantaged adults. Age NS. 76% female. (*n* = 927)	FOODcents nutrition education program: aims to improve household food expenditure according to the healthy eating pyramid, includes food label reading. Delivered face-to-face with cooking sessions and supermarket tours.	Cohort comprising 54% of the FOODcent centers, includes different program durations. Pre-post survey and six-week online follow-up (*n* = 97).	Community-based	Single group session of 1–2 h or up to 8 sessions.	P&P.	Reading of the nutrition information panel (self-reported)	Significantly increased at six-week follow-up (*p* < 0.01).	Knowledge of interpreting food labels used three questions including one item on nutrition labels: “*In 100 g of this product how many grams of sugar are there*?”	Higher proportion of correct responses in post-session surveys. No significant differences by socioeconomic status.
[31] India	School children. Aged 12–15 years. Females: NS. (*n* = 175)	READ-B4-U-EAT multicomponent school module to promote use of food label information and informed food choices. Delivered using videos, handouts and presentations, by teachers.	Intervention group and comparison group using pre-post intervention questionnaires. (Comparison group received a lecture about food labels.)	School	4 sessions of 45 min	SCT	Use of nutrition labels evaluated with 5 questions(self-reported) i.e., “*Do you read the sugar content when buying chocolate*?”	1 question showed improvements in intervention compared to comparison group (*p* < 0.05), i.e., *“Do you see the salt content when buying snacks?”*	Knowledge of nutrition label assessed using item: “*Is nutrition information present on this label*”?	Significant improvement in intervention compared to control group (*p* < 0.05)
[37] U.S.	Low income U.S. adults Aged 18–50 years. 90% female. (*n* = 123)	Web based nutrition education program on healthy eating including nutrition label reading.	Randomized block equivalence (comparator group received in-person taught session).	Own home computer/community centre	3 sessions each 30–40 min	KEL	Frequency of use of labels when shopping (self-reported). “*When shopping do you use nutrition labels to decide what food to buy?*”	Both groups significantly increased at post-intervention but in-person group showed greatest improvement.	NP	NP
[28] U.S.	College students. Aged 17–24 years. 63% females. (*n* = 32)	Thumbs Up Healthy Eating Nutrition Education booklet designed to promote attention focus on nutrition labels on product packaging.	Randomized controlled, pre-posttest. (Control group viewed a word puzzle).	University	A 10-min session.	IP	Eye gaze time on nutrition labels on cereal box packaging images.	Participants in the experimental group gazed longer at nutrition labels during post-test compared to the pre-test (*p* < 0.01) and at posttest compared to the control group (*p* < 0.001).	NP	NP
[35] U.S.	Latinos with Type II diabetes. Median age 57 years. 73% female. (*n* = 203)	Diabetes among Latinos Best Practices Trial (DIALBEST) on food labels and glycemic control. Includes nutrition education and how to interpret food labels. Delivered with individuals by community health workers.	Block-randomized to either intervention or control groups which were evaluated at baseline, 3,6,12,18 months (control group received standard care).	Home-based (and store visit)	17 home-based sessions over a 12-month period.	NS	Frequency of use of food labels (self-reported).	Food label use significantly higher in the intervention vs control groups at 3, 12, and 18 months (*p* < 0.01).	NP	NP
[23] Canada	Adults. Aged 18–65 years. 81% female. (*n* = 259)	Healthy Eating is in Store for You—a nutrition labeling education program aiming to help consumers make food choices promoting healthy weight. Delivered by trained community health officers.	Cohort comprised of 18 workshops across the country. Pre-posttest and 3-month follow-up questionnaires. (*n* = 35)	Community-based	1 session	NS	Nutrition label attitudes and behaviors (self-reported). i.e., “*Is it important to you to review the nutrition information before buying that food”*?	Data on 35 participants only available at 3-month follow-up. Increased proportions of participants selecting higher responses.	NP	NP
[34] UK	Vulnerable adults. Aged >45 years. 68% female. (*n* = 62)	Eat Better Feel Better community-based cooking program aimed at tackling barriers to cooking and healthy eating. Delivered by community-trained chefs.	Single group repeated measures. Pre and post intervention and 3–4 month follow-up (*n* = 17).	Community-based	6-weekly sessions of 2 h.	NS	(1) Confidence reading food labels (self-reported). (2) Food label elements read (indicated using tick boxes)	(1) Significantly increased from baseline to post intervention (*p* < 0.01) (2) Reading of nutrition elements Significantly increased from baseline to post intervention and follow-up.	NP	NP
[32] U.S.	School children in grades 3–5 and 6–8. ~50% female. (*n* = 1334)	Choose Health: Food, Fun, and Fitness Youth Curriculum aimed at enhancing knowledge and skills building. Incudes label reading. Delivered by community health educators.	2 cohort sub-samples, across age groups and settings evaluated using pre-post surveys (which featured nutrition label items)	School, clubs, summer camp	6-weekly lessons 45–90 min each.	SCT EL	Reading of nutrition information (self-reported) i.e., “*I read nutrition facts labels on food packages*”	Significantly increased post-survey (*p* <0.01)	NP	NP
[26] U.S.	School children in grade 3. Mean age 8.7 years. 52% female. (*n* = 1487)	Nutrition Detectives and ABC for Fitness programs (standard intervention), alongside family, home, and supermarket sessions (enhanced intervention).	Quasi-experimental 3 group design. Schools randomized on district. Pre-posttests. (Control group received normal curriculum and no pre-posttests.)	School	90-min class session. 3-month follow-up, 30-min booster.	NS	NP	NP	Food Literacy and Label Nutrition Knowledge (FLLANK) test to evaluate knowledge of healthful food choices.	Both groups increased FLLANK scores compared to baseline values after first and booster sessions (*p <* 0.01). No significant difference in this improvement between the two intervention groups.
[29] U.S.	College students. Mean age 20.7 years. 60% female. (*n* = 140)	Web-based label-reading training tool to improve individuals’ ability to use labels to select more healthful foods. Training tasks required individuals to compare 3 × 24 different pairs of nutrition labels to select the healthiest.	Randomized to 2 groups. Prior knowledge group received short presentation. Basic group did not.	University	One session of 60–90 min.	Skill	NP	NP	Accuracy (of selecting correct answer in training tasks)	Accuracy increased with practice, across each of the three training blocks (*p* < 0.01). In block 3, the odds of a correct answer for the prior-knowledge group were 79% higher than those in the basic group (*p* = 0.02).
[33] U.S.	Young adolescents. Aged 11–14 years. 47% female. (*n* = 34)	How to read and use a nutrition facts label education program. Delivered by a registered dietitian.	Single cohort using pre-posttests.	NS	1 group session of 1 h.	NS	NP	NP	Nutrition Facts Label knowledge pre- posttests developed by author (calculating %DV with differing serving sizes and defining DV).	Overall test score improved significantly pre-posttest (*p* < 0.01) Correct answers to the questions concerning %DV definition improved significantly (*p* = 0.03) from 38% to 74%, as did correct answers to question concerning serving size modification calculations (*p* = 0.003). No difference in boys or girls scores.
[30] U.S.	Grade 5 school children. Age NS. 58% female. (*n* = 212)	Nutrition Detectives educational program on how to read food labels aimed at developing food-literacy skills. Taught by teacher within class (presentation and practical)	Cohort comprising of classes across 5 schools, using pre -posttests.	School class	1 session of 45 min	NS	NP	NP	Food label literacy (quiz) evaluating ability to distinguish between healthy and unhealthy foods using the Nutrition Facts panel.	Quiz scores increased significantly pre-posttest by 16.2% (ranging from 4.3%–23.6% among schools) (*p* < 0.01). Girls score improved significantly more than boys (*p* = 0.04)

NS = not stated; NP = not performed; NFP = Nutrition Facts Panel; SCT = Social Cognitive Theory; TML = Theory of Meaningful Learning; SoC = Stages of Change; Skill = skills acquisition, KEL = Kolb’s experiential learning; EL = experiential learning; IP=information processing; P&P = precede and proceed; % DVs = % daily values.

## Supplementary Materials

The following are available online at http://www.mdpi.com/2072-6643/10/10/1432/s1. Table S1: Search terms and strategy for the Medline database. Table S2: Quality appraisal of individual studies using Effective Public Health Practice Project (EPHPP) Criteria. S1: PRISMA 2009 checklist.
