# Supplementary material for: Effect of Educational Interventions on Understanding and Use of Nutrition Labels: A Systematic Review
Source: Nutrients. 2018 Oct 4;10(10):1432. doi: 10.3390/nu10101432 (PMC6213388; doi:10.3390/nu10101432)
Supplement: Supplementary file 1 [file nutrients-10-01432-s001.pdf]

Table S1 Search terms and strategy for Medline database.

	Search Term		Search Term
1	nutrition* label*.	21	Health Promotion/
	Food Labeling/	22	(health adj3 promot*).mp. [
3	food label*.	23	(health adj2 educat*).mp
4	(nutrition* adj3 information).mp.	24	14 or 15 or 16 or 17 or 18 or 19 or 20 or 21 o 22 or 23 or "24".mp.
5	(nutrition* facts panel or nutrition* facts table).mp	25	cognition/ or awareness/ or comprehension/knowledge
6	food packag*.mp.	26	understand*.mp.
7	exp Nutritional Sciences/	27	perception.mp.
8	Nutritional Status/	28	comprehen*.mp
9	Nutrition Labels/	29	consumer*.mp.
10	Nutritive value/	30	health literacy.mp.
11	nutriti* value.mp.	31	nutrition literacy.mp.
12	food* value.mp.	32	behavio*.mp.
13	1 or 2 or 3 or 4 or 5 or 6 or 7 or 8 or 9 or 10 or 11 or 12	33	behavio* change.mp.
14	health education.mp.	34	Consumer behavior/
15	Health Education/	35	Consumer behaviour/
16	education* intervention* program*.mp	36	Health behaviour/
17	nutrition education food label*.mp.	37	25 or 26 or 27 or 28 or 29 or 30 or 31 or 32 o 33 or 34 or 35 or 36
18	(nutrition* adj3 education).mp.	38	13 and 24 and 37
19	Patient Education as Topic/		
20	Health Knowledge, Attitudes, Practice/ or Program Evaluation/		

Table S2 Quality Appraisal of included studies using EPHPP Criteria.

Reference number	Selection bias	Study design	Confounders	Blinding	Data collection	Withdrawals/dropouts	Intervention integrity	Overall
Randomized studies								
27	Strong	Strong	Strong	Weak	Strong	Strong	Moderate	Strong
25	Strong	Strong	Strong	Weak	Strong	Strong	Moderate	Strong
38	Moderate	Strong	Strong	Weak	Strong	Strong	Moderate	Strong
37	Strong	Strong	Strong	Weak	Moderate	Strong	Moderate	Moderate
28	Moderate	Moderate	Weak	Weak	Strong	Weak	Moderate	Moderate
35	Strong	Strong	Strong	Moderate	Moderate	Moderate	Moderate	Strong
26	Strong	Moderate	Moderate	Weak	Strong	Strong	Moderate	Moderate
29	Strong	Strong	Moderate	Moderate	Strong	Strong	Strong	Strong
31	Strong	Moderate	Moderate	Moderate	Weak	Strong	Moderate	Moderate
Cohort studies								
33	Moderate	Weak	Weak	Moderate	Moderate	Moderate	Moderate	Moderate
30	Moderate	Weak	Weak	Moderate	Strong	Moderate	Moderate/ weak	Moderate
39	Moderate	Moderate	Moderate	Weak	Strong	Weak	Moderate /weak	Moderate
23	Weak	Weak	Weak	Weak	Moderate	Moderate	Weak	Weak
24	Weak	Moderate	Weak	Weak	Weak	Weak	Moderate	Weak
34	Strong	Moderate	Moderate	Moderate	Strong	Weak	Moderate	Moderate
32	Strong	Moderate	Moderate	Moderate	Moderate	Weak	Moderate	Moderate
36	Strong	Moderate	Moderate	Moderate	Strong	Weak	Moderate	Moderate

Supplementary S1 PRISMA 2009 checklist.

Section/topic	#	Checklist item	Reported on page #
**TITLE**			
Title	1	Identify the report as a systematic review, meta-analysis, or both.	1
**ABSTRACT**			
Structured summary	2	Provide a structured summary including, as applicable: background; objectives; data sources; study eligibility criteria, participants, and interventions; study appraisal and synthesis methods; results; limitations; conclusions and implications of key findings; systematic review registration number.	1
**INTRODUCTION**			
Rationale	3	Describe the rationale for the review in the context of what is already known.	1-2
Objectives	4	Provide an explicit statement of questions being addressed with reference to participants, interventions, comparisons, outcomes, and study design (PICOS).	2-3
**METHODS**			
Protocol and registration	5	Indicate if a review protocol exists, if and where it can be accessed (e.g., Web address), and, if available, provide registration information including registration number.	n/a
Eligibility criteria	6	Specify study characteristics (e.g., PICOS, length of follow-up) and report characteristics (e.g., years considered, language, publication status) used as criteria for eligibility, giving rationale.	3
Information sources	7	Describe all information sources (e.g., databases with dates of coverage, contact with study authors to identify additional studies) in the search and date last searched.	2-3
Search	8	Present full electronic search strategy for at least one database, including any limits used, such that it could be repeated.	Table S1 page 16. Page 2.
Study selection	9	State the process for selecting studies (i.e., screening, eligibility, included in systematic review, and, if applicable, included in the meta-analysis).	2-3
Data collection process	10	Describe method of data extraction from reports (e.g., piloted forms, independently, in duplicate) and any processes for obtaining and confirming data from investigators.	3
Data items	11	List and define all variables for which data were sought (e.g., PICOS, funding sources) and any assumptions and simplifications made.	3

Risk of bias in individual studies	12	Describe methods used for assessing risk of bias of individual studies (including specification of whether this was done at the study or outcome level), and how this information is to be used in any data synthesis.	3 (Table S2 page 17)
Summary measures	13	State the principal summary measures (e.g., risk ratio, difference in means).	2-3
Synthesis of results	14	Describe the methods of handling data and combining results of studies, if done, including measures of consistency (e.g., I^2) for each meta-analysis.	2-3

Section/topic	#	Checklist item	Reported on page #
Risk of bias across studies	15	Specify any assessment of risk of bias that may affect the cumulative evidence (e.g., publication bias, selective reporting within studies).	15-16
Additional analyses	16	Describe methods of additional analyses (e.g., sensitivity or subgroup analyses, meta-regression), if done, indicating which were pre-specified.	n/a
**RESULTS**			
Study selection	17	Give numbers of studies screened, assessed for eligibility, and included in the review, with reasons for exclusions at each stage, ideally with a flow diagram.	2--4 (and Figure 1 flow chart page 4)
Study characteristics	18	For each study, present characteristics for which data were extracted (e.g., study size, PICOS, follow-up period) and provide the citations.	4-10 (Table 1)
Risk of bias within studies	19	Present data on risk of bias of each study and, if available, any outcome level assessment (see item 12).	2-3 (table S2 page 17)
Results of individual studies	20	For all outcomes considered (benefits or harms), present, for each study: (a) simple summary data for each intervention group (b) effect estimates and confidence intervals, ideally with a forest plot.	Table 1 4-10
Synthesis of results	21	Present results of each meta-analysis done, including confidence intervals and measures of consistency.	n/a
Risk of bias across studies	22	Present results of any assessment of risk of bias across studies (see Item 15).	n/a
Additional analysis	23	Give results of additional analyses, if done (e.g., sensitivity or subgroup analyses, meta-regression [see Item 16]).	n/a
**DISCUSSION**			

Summary of evidence	24	Summarize the main findings including the strength of evidence for each main outcome; consider their relevance to key groups (e.g., healthcare providers, users, and policy makers).	11-13
Limitations	25	Discuss limitations at study and outcome level (e.g., risk of bias), and at review-level (e.g., incomplete retrieval of identified research, reporting bias).	13-14
Conclusions	26	Provide a general interpretation of the results in the context of other evidence, and implications for future research.	14-15
**FUNDING**			
Funding	27	Describe sources of funding for the systematic review and other support (e.g., supply of data); role of funders for the systematic review.	15

From: Moher D, Liberati A, Tetzlaff J, Altman DG, The PRISMA Group (2009). Preferred Reporting Items for Systematic Reviews and Meta-Analyses: The PRISMA Statement. PLoS Med 6(7): e1000097. doi:10.1371/journal.pmed1000097.

For more information, visit: **www.prisma-statement.org**.
